# COVID-19 hospitalization in vaccinated and non-vaccinated patients: Clinical profile and outcomes

**DOI:** 10.1016/j.bjid.2025.104537

**Published:** 2025-05-09

**Authors:** Laura Holtman-Ferreira, Elessandra de Souza Bitencourt, Betina Mendez de Alcantara Gabardo, Susanne Edinger Pereira, Francine Teixeira, Diego da Silva Magatão, Vitor Loureiro Dias, Ricardo Petterle, Meri Bordignon Nogueira, Sonia Mara Raboni

**Affiliations:** a Universidade Federal do Paraná (UFPR), Programa de Pós-Graduação em Microbiologia, Parasitologia e Patologia, Curitiba, PR, Brazil; b Universidade Federal do Paraná (UFPR), Complexo Hospital das Clínicas, Centro de Pesquisa da Unidade de Doenças Infecciosas, Curitiba, PR, Brazil; c Universidade Federal do Paraná (UFPR), Complexo Hospital das Clínicas, Unidade de Doenças Infecciosas, Curitiba, PR, Brazil; d Universidade Federal do Paraná (UFPR), Complexo Hospital das Clínicas, Unidade de Pneumologia, Curitiba, PR, Brazil; e Universidade Federal do Paraná (UFPR), Departamento de Medicina Integrativa, Curitiba, PR, Brazil

**Keywords:** SARS-CoV-2, Vaccine, Immune response, Immunization, Public health, Pandemic

## Abstract

COVID-19, caused by SARS-CoV-2 infection, left widespread impacts worldwide. In Brazil, immunization reduced incidence rates. However, six months later, waning neutralizing antibody titers and new immune-evading variants increased cases, resulting in recurring waves. This study evaluated hospitalized COVID-19 patients after the vaccination rollout, comparing the clinical outcomes between vaccinated and unvaccinated patients. Positive samples underwent nucleotide sequencing. A total of 218 patients were included; 202 (92 %) had vaccination data, 98 received at least one dose, and 64 completed the vaccination schedule, predominantly with CoronaVac®. Vaccinated individuals were older on average since the campaign was primarily conducted among the elderly. The Gamma variant predominated during the study period. While not statistically significant, trends indicated greater respiratory assistance needs, more extended hospital stays, and increased ICU time among unvaccinated patients. Mortality was 45 % in vaccinated and 37 % in unvaccinated groups, with no notable difference. However, patients with a complete vaccination schedule showed a higher chance of survival, though not significant (*p* = 0.11). The factors significantly associated with higher mortality were older patients, those requiring vasopressor drugs, and mechanical ventilation. These findings provide clinical, epidemiological, and phylogenetic insights into COVID-19 patients during vaccination implementation. They underscore the need to evaluate vaccine effectiveness against circulating variants and highlight the importance of complete vaccination schedules for improving patient outcomes.

## Introduction

In 2019, following its initial identification in Wuhan, China, SARS-CoV-2 rapidly evolved into a pandemic pathogen, spreading across multiple continents and triggering a profound global impact.^1^

In Brazil, the vaccination campaign began on a large scale, with four vaccines developed using different platforms. The nationwide SARS-CoV-2 immunization program was launched in January 2021 and was gradually rolled out, initially prioritizing older adults, healthcare professionals, and individuals at high risk of severe disease. Subsequently, it expanded to include the entire adult population. Even though antivirals are available in the public health system for outpatients and hospitalized patients who meet clinical criteria, vaccination remains the most effective prevention strategy.^2^^,^^3^

In Brazil, the first vaccine utilized to control the pandemic was CoronaVac® (Sinovac Life Sciences, Beijing, China), based on the inactivated viruses’ platform, followed by Comirnaty® (BNT162b2, RNAm, Pfizer-BioNTech, USA), ChAdOx1 nCoV-19 (AZD1222, Oxford/AstraZeneca, UK), and Ad26.COV2.S (Johnson &. Johnson–Janssen). As a result of the slow and gradual implementation of the immunization program and the surge of viral variants with mutations and deletions in the S protein sites that enhance viral entry, replication, and immune evasion, the vaccines have been less effective in preventing hospitalization.^4^

This scenario, combined with the decline in antibody titer observed six months after vaccination, raises concerns about the long-term protection of the immunized population, posing a significant public health challenge. Currently, the vaccination schedule includes multiple boosters for all ages, totaling >500 million doses administered (https://infoms.saude.gov.br/extensions/SEIDIGI_DEMAS_Vacina_C19/SEIDIGI_DEMAS_Vacina_C19.html). To this day, little is known about the long-term effectiveness of COVID-19 vaccines in reducing the severity and mortality associated with the currently circulating variants. This uncertainty persists despite the availability of antiviral therapies and multiple reinfections among patients. This study aimed to describe the epidemiological, clinical profiles, and outcomes of a hospitalized cohort of SARS-CoV-2 infected patients and assess whether vaccination was a protective factor against severe disease during the emergence of Gamma and Delta variants of concern.

## Material and methods

### Study design

This retrospective cohort study was conducted with hospitalized patients diagnosed with COVID-19 at the Clinical Hospital Complex of the Federal University of Paraná (CHC-UFPR), a tertiary academic hospital in southern Brazil. Patients had been previously screened and included in the multicentric study “The effectiveness of COVID-19 vaccines in Latin America, 2021: a multicenter regional case-control study”, supported by the Pan American Health Organization (PAHO) and Oswaldo Cruz Institute (Fiocruz). The Institutional Ethics Board revised and approved this study (n° 53,891,121.0.0000.0096).

The inclusion criteria were hospitalized patients between August 2021 and February 2022 who were ≥ 18-years-old, eligible for the anti-SARS-CoV-2 vaccine, presenting cough and fever, with at least 3-days of symptoms, and had not been infected with SARS-CoV-2 in the past 90-days. Patients with no clinical data, contraindication to the vaccine, or unconfirmed SARS-CoV-2 infection were excluded.

### Data and samples

The researchers obtained retrospective clinical, epidemiological, vaccination status, and outcome data from electronic medical charts. Data collection encompassed the entirety of the patient’s hospitalization, concluding at the time of discharge or death.

Patients were categorized by vaccination status: vaccinated and non-vaccinated, complete and incomplete vaccination schedules, and timely and untimely vaccination for those fully vaccinated. Timely vaccination was defined as receiving the vaccination at least 14-days before the infection. A compliance group was used to identify those patients who had received full and timely vaccine doses.

After diagnosis, swab samples with sufficient volume and a SARS-CoV-2 qRT-PCR with Ct < 30 were sent to the Respiratory Viruses and Measles Laboratory (LVRS, Fiocruz, Rio de Janeiro, Brazil) for complete genome sequencing. The Fiocruz Genomic Network performed the analysis using the *ViralFlow pipeline* (Available at: https://viralflow.github.io/). Data on circulating VOCs were obtained from the Genomic Surveillance of SARS-CoV-2 in Brazil. (SESA-PR, 2021, available at: https://www.saude.pr.gov.br/Pagina/Coronavirus-COVID-19).

### Phylogenetic analysis

To examine the evolution and prevalence of SARS-CoV-2 strains in admitted patients, the complete genome sequences generated in this study were compared with genome sequences from reference strains identified in Brazil. These sequences were retrieved from GISAID (https://gisaid.org/) until December 2022. All sequences were aligned using the MAFFT program (Available at: https://mafft.cbrc.jp/) and manually edited to remove artifacts such as insertions, deletions, and gaps using the Aliview program (Available at: https://github.com/NCIP/alview). The Maximum Likelihood (ML) phylogenetic trees were estimated using IQ-TREE2 (Available at: http://www.iqtree.org/), using the GTR nucleotide substitution model, and the resulting tree was edited using FigTree (V.1.4.3, available at http://tree.bio.ed.ac.uk/software/figtree/).

### Data analysis

Data were analyzed using *R* Core Team Statistical Software, V.4.2.1 (R Foundation for Statistics Computing, Vienna, Austria, 2014). Relative and absolute frequencies were analyzed, and Fisher's exact, Chi-Square, Mann-Whitney, and Gray tests were applied, as appropriate. An unadjusted bivariate analysis was performed comparing fully vaccinated patients with unvaccinated or partially vaccinated patients and comparing death and discharge outcomes. Multivariate analysis to assess disease severity was performed, considering that those with bivariate analysis results had presented a p-value < 0.2 as explanatory variables. All p-values are based on two-tailed comparisons, and the significance level was set at *p* < 0.05.

The Kaplan-Meier method compared the case survival curves according to the vaccination schedule. The log-rank test was used to verify differences between groups. Survival curves were stratified by the presence of vaccination and by the completeness of the vaccination schedule.

## Results

**Fig. 1:**
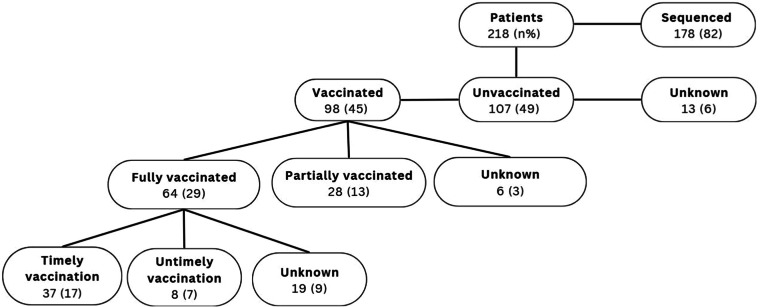
Flowchart of study design.

A total of 218 participants were initially included. Of these, 202 (92 %) had vaccination data available, with 98 vaccinated and 107 unvaccinated patients (Fig. 1). Among the vaccinated patients, only 6 (3 %) did not have data on the vaccination scheme used. In addition, some medical records reported a complete vaccination schedule before admission, but the vaccine date was not informed, hindering the ability to evaluate the timeliness of immunization.

Table 1 compares the clinical and demographic profiles of vaccinated (45 %) and non-vaccinated (49 %) patients. For both groups, most patients were male, had completed primary school, and never smoked. The most common symptoms were dyspnea (70 %, 69 %) and cough (58 %, 51 %) for vaccinated and unvaccinated patients, respectively. Regarding the vaccinated ones, more than half (60 %) received the CoronaVac® vaccine (Sinovac Biotech, Bejin, China). The second vaccine most frequently administered beyond the analyzed sample was the ChAdOx1 nCoV-19 vaccine (27), followed by Comirnaty® (9) and Ad26. COV2.S.^1^ Gamma and Delta VOCs were the most frequent viral genotypes found. Unvaccinated patients were significantly younger. There was a trend towards a higher need for respiratory support, longer length of hospitalization, and increased ICU time among the unvaccinated group, although the difference was not statistically significant. There was no significant difference in the mortality rate between the vaccinated and unvaccinated groups, with rates of 45 % and 37 %, respectively.

**Table 1:** Clinical, epidemiological, and outcome characteristics of the vaccinated and unvaccinated groups, and among the vaccinated, according to compliance with the vaccine schedule.

Epidemiological and clinical characteristics	Vaccinated		*p value* Unajusted analysis	Compliance		*p value* Unajusted analysis
	Yes *n* = 98(%)	No *n* = 107 (%)		Yes *n* = 64 (%)	No *n* = 28 (%)	
Sex, n						
Male, n	52 (53)	58 (54)	0.8893	32 (50)	16 (57)	0.6510
Age, y						
Median (IQR)	69 (57.75, 78)	53 (40, 67)	**< 0.0001**	73 (66, 79)	63 (52, 73)	0.0589
Smoking habit, n						
Smoker	13 (13)	6 (6)	0.0896	8 (12)	3 (11)	1
Past smoker	24 (24)	24 (22)	0.744	19 (30)	5 (18)	0.306
Never smoked	29 (29)	28 (26)	0.6409	22 (34)	6 (21)	0.3246
Educational level, n						
Illiterate	5 (5)	1 (0.9)	0.1058	2 (3)	3 (11)	0.1629
Elementary school	43 (44)	47 (44)	1	26 (41)	15 (53)	0.2649
High School	15 (15)	23 (21)	0.2839	10 (16)	4 (14)	1
University degree	4 (4)	4 (4)	1	3 (5)	1(3)	1
Symptoms at admission						
Fever	38 (39)	40 (37)	0.0122	26 (41)	8 (28)	0.3498
Cough	57 (58)	55 (51)	**< 0.0001**	38 (59)	15 (53)	0.651
Weakness/ fatigue	40 (41)	30 (28)	0.0572	26 (41)	11 (39)	1
Headache	17 (17)	13 (12)	0.3269	13 (20)	2 (7)	0.1378
Myalgia	28 (28)	23 (21)	0.261	19 (30)	7 (25)	0.8042
Sore throat	8 (8)	11 (10)	0.638	6 (9)	2 (7)	1
Runny nose	7 (7)	8 (7)	1	5 (8)	1(3)	0.6632
Anosmia and ageusia	12 (12)	10 (9)	0.6523	9 (14)	0	0.0528
Dyspnea	69 (70)	74 (69)	0.8799	9 (14)	25 (90)	< 0.0001
Anorexia/nausea / vomiting	28 (28)	23 (21)	0.261	9 (14)	4 (14)	1
Diarrhea	11 (11)	12 (11)	1	9 (14)	2 (7)	0.4936
Altered mental state	21 (21)	25 (23)	0.8671	9 (14)	9 (32)	0.0836
Vaccine doses						
Completed Schedule	64 (65)					
Timely vaccination	37 (38)					
Vaccine type						
CoronaVac (Sinovac®)	59 (60)			47 (73)	9 (32)	**0.0004**
Comirnaty (Pfizer®)	9 (9)			3 (5)	6 (21)	0.0211
ChAdOx1 (Astrazeneca®)	27 (27)			11 (17)	13 (46)	**0.0049**
Ad26.COV2.S (Janssen®)	1 (1)			1 (1)	0	1
Nucleotide Sequenced samples and VOCs detected	76 (77)	90 (84)	0.2857	51 (80)	21 (75)	0.5963
Gamma	40 (41)	75 (70)	**< 0.0001**	19 (30)	20 (71)	**0.0003**
Delta	36 (37)	1 (0.9)	**< 0.0001**	32 (50)	1(3)	**< 0.0001**
Zeta	0	11 (10)	**0.0008**	0	0	
Alpha	0	3 (3)	0.2478	0	0	
Admission data						
Days of symptoms at admission, mean ±SD	6 (2.8)	4 (3.8)	0.5062	6 (2.8)	5 (2.8)	0.8215
Other clinical and laboratory data						
Abnormal thoracic images, n	84 (86)	97 (91)	0.2864	52 (81)	27 (96)	0.0997
Oxygen saturation < 94 %, n	97 (99)	106 (99)	1	63 (98)	28 (100)	1
Tachypnea (RF > 30), n	46 (47)	43 (40)	0.0843	28 (44)	16 (57)	0.0759
Pulmonary infiltrates > 50 %, n	30 (31)	37 (34)	0.5552	15 (23)	14 (50)	0.0155
Use of vasoactive drugs	40 (41)	45 (42)	0.8878	20 (31)	18 (62)	0.0053
Dialysis, n	13 (13)	17 (16)	0.6936	6 (9)	6 (21)	0.1755
Intensive care unit needed	58 (59)	63 (59)	1	34 (53)	22 (78)	0.0355
Need for respiratory support	94 (96)	107 (100)	0.0506	60 (94)	28 (100)	0.3098
Spontaneous ventilation	55 (56)	46 (43)	0.0697	42 (66)	9 (32)	**0.0057**
Mechanical ventilation	39 (40)	56 (52)		18 (28)	19 (68)	
Days of ICU, median (IQR)	8 (4, 12)	14 (5, 18)	0.0788	8 (4, 11)	7 (4, 16)	0.57
Presence of Comorbidities	87 (89)	87 (81)		57 (89)	25 (90)	
Cardiovascular diseases	67 (68)	61 (57)	0.1126	45 (70)	19 (68)	0.8103
Neurological diseases	17 (17)	14 (13)	0.4387	10 (16)	7 (25)	0.3814
Lung diseases	24 (24)	19 (18)	0.3029	16 (25)	6 (21)	0.7956
Gastrointestinal diseases	7 (7)	7 (6)	1	4 (6)	3 (11)	0.4314
Endocrine diseases	46 (47)	37 (34)	0.0807	30 (47)	13 (46)	1
Kidney disease	14 (14)	9 (8)	0.0098	10 (16)	3 (11)	0.7474
Hematological diseases	11 (11)	6 (6)	0.2048	6 (9)	4 (14)	0.4857
Malignancies	15 (15)	5 (5)	**0.0167**	11 (17)	4 (14)	1
Immunodeficiency	10 (10)	14 (13)	0.6644	6 (9)	2 (7)	1
Psychiatric treatment	6 (6)	11 (10)	0.3198	5 (8)	1(3)	0.6632
Other infectious diseases	4 (4)	0	0.0506	3 (5)	0	0.5505
Obesity	34 (35)	26 (24)	0.1762	23 (36)	7 (25)	0.3434
Outcome						
Death	44 (45)	40 (37)	0.3214	23 (36)	19 (68)	0.0063

SD, Standard Deviation; IQR, Interquartile Range. In bold: statistically significant p-values.

Out of 98 vaccinated patients, 92 confirmed the completion of immunization. Of these, 70 % (65/92) adhered to the recommended vaccination schedule (compliance), which included one dose of Ad26.COV2.S and two doses of another vaccine (Table 1). Dyspnea was the most frequent symptom, reported by90 % of the non-compliance group, which also had a significantly higher mortality rate compared to the compliance group (68 % vs. 36 %, *p* = 0.0063).

**Fig. 2:**
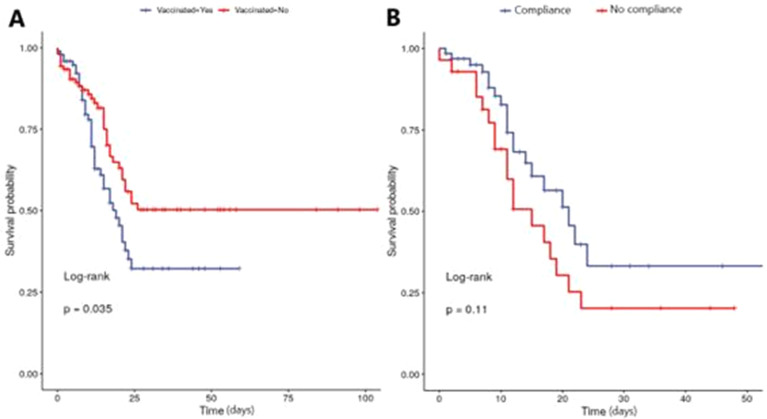
Survival curve, comparing vaccinated and non-vaccinated patients (A) and vaccinated patients with compliance and non-compliance to the vaccine scheme (B).

A survival curve was built to examine the impact of immunization between the different groups. When analyzing the vaccinated and unvaccinated groups, the chance of survival was significantly higher among the unvaccinated ones (Fig. 2A) (*p* = 0.035), and patients with a complete vaccination schedule had a greater chance of survival (Fig. 2B), but the difference was not significant (*p* = 0.11).

Subsequently, a comparison was made between patients according to clinical outcomes, discharge or death. The results are presented in Table 2. In the adjusted analysis, the factors associated with the outcome of death were older age, the need for vasopressor drugs, and mechanical ventilation.

**Table 2:** Correlation between clinical and epidemiological findings and outcomes.

Epidemiological and clinical findings	Discharge *n* = 122 (%)	Death *n* = 95 (%)	Unadjusted analysis *p value*	Adjusted analysis* OR (95 % CI)
Sex				
Male, n	58 (47)	56 (59)	0.06214	NS
Age, y				
Median (IQR)	57 (17, 42)	69 (17, 53)	< 0.001	1.05 (1.03 – 1.09)
Smoking habit				
Smoker	9 (7)	13 (14)	0.09423	NS
Former smoker	34 (28)	16 (17)		
Never smoked	38 (31)	23 (24)		
Educational level				
Illiterate	5 (4)	2 (2)	0.5298	
Elementary school	51 (42)	41 (43)		
High School	14 (11)	14 (15)		
University degree	6 (5)	2 (2)		
Symptoms at admission				
Fever	52 (43)	29 (30)	0.3154	
Cough	70 (57)	46 (48)	1.00	
Weakness/ fatigue	41 (34)	35 (37)	0.0353	
Headache	23 (19)	7 (7)	0.3074	
Myalgia	39 (32)	15 (16)	0.6639	
Sore throat	13 (11)	6 (6)	0.3715	
Runny nose	13 (11)	2 (2)	0.122	
Anosmia and ageusia	18 (15)	4 (4)	0.1232	
Dyspnea	79 (65)	77 (81)	0.01229	NS
Anorexia/nausea / vomiting	34 (28)	21 (22)	0.08393	
Diarrhea	16 (13)	7 (7)	1.00	
Altered mental state	21 (17)	31 (32)	0.0017	NS
Vaccine doses				
Unvaccinated	66 (54)	17 (18)		
Completed Schedule	41 (34)	23 (24)	0.0093	NS
Timely vaccination	23 (19)	14 (15)	1.00	
Vaccine type				
CoronaVac (Sinovac®)	31 (25)	28 (29)	0.345	
Comirnaty (Pfizer®)	3 (2)	6 (6)		
ChAdOx1 (Astrazeneca®)	17 (14)	10 (10)		
Ad26.COV2.S (Janssen®)	3 (2)	0 (0)		
Variants of Concern (VOCs)				
Gamma	60 (49)	65 (68)	0.0064	
Delta	28 (23)	9 (9)		
Zeta	7 (6)	5 (5)		
Alpha	3 (2)	0 (0)		
Admission data				
Days of symptoms at admission ±SD	6 (3, 9)	5 (1, 8)	0.1174	
Other Clinical and Laboratory findings				
Abnormal thoracic images	107 (88)	82 (86)	1.00	
Oxygen saturation < 94 %	121 (99)	95 (100)	0.506	
Tachypnea (RR > 30)	39 (32)	59 (62)	<0.001	NS
Pulmonary infiltrates > 50 %	33 (27)	37 (39)	0.08824	NS
Use of vasoactive drugs	17 (14)	76 (80)	<0.001	9.76 (3.64 – 27.91)
Dialysis	5 (4)	28 (29)	<0.001	NS
Intensive care unit needed	42 (34)	87 (91)	<0.001	NS
Need for respiratory support	114 (93)	95 (100)	0.00553	
Spontaneous ventilation	89 (73)	16 (17)	<0.001	7.02 (2.48 – 20.56)
Mechanical ventilation	25 (20)	79 (83)		
Days of ICU	9 (5,21)	8 (5, 15)	0.1932	
Presence of Comorbidities	97 (97)	89 (93)		
Cardiovascular diseases	71 (58)	66 (69)	0.1012	
Neurological diseases	16 (13)	16 (17)	0.5267	
Lung diseases	22 (18)	26 (27)	0.1202	
Gastrointestinal diseases	10 (8)	4 (4)	0.2805	
Endocrine diseases	49 (40)	40 (42)	0.792	
Kidney disease	13 (10)	12 (12)	0.7736	
Hematological diseases	10 (8)	7 (7)	1.00	
Malignancies	10 (8)	11 (11)	0.5155	
Immunodeficiency	15 (12)	10 (10)	0.8876	
Psychiatric treatment	13 (10)	6 (6)	0.4016	
Other infectious diseases	4 (3)	0 (0)	0.2	
Obesity	38 (31)	25 (26)	1.00	

IQR, Interquartile Range; RR, Respiratory Rate; ICU, Intensive Care Unit; NS, Non-Significant result.

^a^Only values with statistically significant results are presented.

**Fig. 3:**
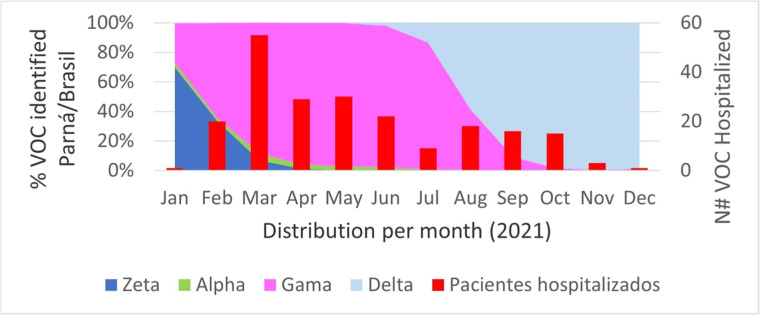
Distribution of VOCs identified by month in 2021.

All 218 admissions analyzed in this study were ordered by month. Fig. 3 compares these admissions to the distribution of Variants of Concern (VOCs) identified at the time of the survey in Paraná.

**Fig. 4:**
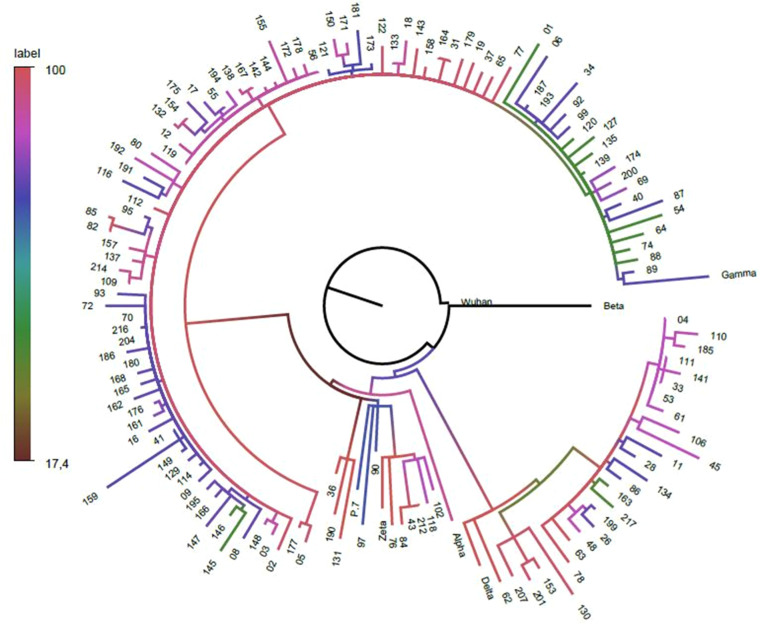
Phylogenetic tree of SARS-CoV-2 strains from admitted patients. Note: Phylogenetic trees based on maximum-likelihood analysis were built using complete sequences of SARS-CoV-2 strains from patients hospitalized with COVID-19 between August 2021 and February 2022. The black dots at the end of the branches represent taxa. The study’s sequences are numbered, and reference sequences are identified in the figure and are available in Supplementary Material.

The sequencing results were compiled and are shown as a phylogenetic tree (Fig. 4). The Gama variant was the predominant VOC among Delta, Alpha, and Zeta variants during the study period.

## Discussion

The rapid development of vaccines using various platforms has been the primary strategy in fighting SARS-CoV-2. However, low- and middle-developed countries faced restricted availability of vaccines with higher efficacy.^5^ Our study examined the outcomes of vaccinated patients compared with non-vaccinated patients hospitalized with COVID-19 a few months after starting anti-COVID-19 vaccination and showed no significant differences between the groups. Factors including adherence to vaccination schedules and the emergence of Variant of Concern (VOC) strains may have influenced these results.

Brazil faced increasing challenges in vaccine adherence, stemming from concerns about vaccine efficacy and difficulties in identifying SARS-CoV-2 infections, particularly in older adults. These issues heightened underreporting and vaccine hesitancy, as some segments of the population were hesitant to recognize the severity of the infection and the advantages of vaccination. Consequently, these factors influenced hospital admissions and vaccination data trends.

Studies have shown that individuals vaccinated for >6-months have a critical reduction in antibody titers.^6^ However, demographic and individual factors can affect the immune response to the immunogenic stimulus. This, combined with extensive viral genetic variability, results in the frequent emergence of viral variants. Therefore, ongoing studies must assess the relationship between vaccination, SARS-CoV-2 infection, and disease severity.^7^

When comparing the vaccinated and unvaccinated groups, only the age variable remained statistically different after the adjusted analysis. Specifically, older individuals had a greater likelihood of being vaccinated. This finding reflects the Brazilian vaccination scenario, where the vaccination campaign began in January 2021 and followed a list of priority groups for vaccination. Older adults, due to the immunosenescence and a higher prevalence of comorbidities, were given priority for the anti-SARS-CoV-2 vaccine, along with healthcare professionals, who were at greater risk of infection due to more frequent exposure.^8^^,^^9^

Regarding sociodemographic data, some reports indicate that it is essential to identify risk or protective factors, such as male sex, dark or brown skin, obesity, and other comorbidities. However, we could not observe the influence of these factors on the patient's outcomes.^10^

The epidemiological profile of patients with a compliance and noncompliance vaccination schedule was similar to the vaccinated and unvaccinated patients. Age continued to be a statistically significant variable, which is in line with the Brazilian scenario at the time of data collection. Patients with incomplete vaccination showed slightly more severe symptoms, including dyspnea, fatigue, muscle weakness, and altered mental status. However, desaturation and the need for supplemental oxygen were common in both groups. Notably, CHC-UFPR, as a tertiary reference hospital, frequently manages moderate to severe cases.

Among patients with a complete vaccination schedule, discharge was the most frequent outcome, while among patients with an incomplete vaccination schedule, the most frequent outcome was death. For these groups, age, type of vaccine, VOCs identified, and outcome were the most significant variables in the bivariate analysis. However, only age remained statistically significant after adjustment.

The Gamma VOC was the most prevalent compared to Zeta, Delta, and Alpha. Reports from respiratory virus surveillance in Curitiba showed alignment between the phylogenetic tree distribution and the circulating variants identified in the city. While Gamma is often associated with greater disease severity, consistent with our findings and a previous analysis conducted by our group, we could not demonstrate an increase in disease severity linked to Gamma or other VOCs.^11^

The survival curve did not show a statistically significant difference between vaccinated and non-vaccinated patients. In the Brazilian context, CoronaVac was the most utilized vaccine in the study population. A subsequent multicenter vaccine effectiveness study conducted in Latin America, which included data from the Curitiba Center (Brazil), reported that all vaccines administered in Brazilian territory showed effectiveness. However, there was variability between products.^9^ As expected, vaccine effectiveness was greater in patients with a complete vaccination schedule. CoronaVac had an efficacy rate of 53 % in this study in patients with a complete vaccination schedule. Furthermore, the vaccine's effectiveness decreased with advancing age.^12^ Given that the majority of participants in this study are mostly older individuals, it is possible that the vaccine effectiveness was reduced due to a decline in immunity, reinforcing the need to administer booster doses.^6^^,^^13^, ^14^, ^15^

Booster doses are recommended in homologous or heterologous modalities and have positive effects in increasing immunity, including against VOCs, which have emerged after the development of vaccines that targeted the infection of the ancestral strain (Wuhan).^16^ Furthermore, a single dose of CoronaVac was associated with low protection in symptomatic patients and hospital admissions, highlighting the need for a complete vaccination schedule.^17^

To understand the higher probability of survival among unvaccinated patients, an analysis of fatal cases revealed that vaccinated patients who had not developed serological immunity before becoming infected with the COVID-19 virus were more likely to die (Suppl. data ‒ Fig. 4). Another study at CHC-UFPR analyzing the serum of healthcare professionals at the hospital to identify anti-S1 and anti-N IgG antibodies as a tool to predict neutralization found a seroconversion rate of 97 % among HCWs 40-days after receiving the first dose.^6^ In contrast to other vaccines in Brazil, CoronaVac showed low protection until the second dose was administered (over four weeks after the first dose) .^17^ Ranzini and colleagues recommended prioritizing full vaccination schedules over single doses for better protection, especially for older adults.

Additionally, Lira et al. reported similar findings, showing more severe infections in individuals who had not completed the vaccination schedule or were not vaccinated at all. However, unvaccinated patients still showed a 26 % lower chance of dying. These results could be influenced by factors such as underreporting during the pandemic, the slow progress of vaccination in the country, and vaccine hesitancy among the population, all of which may have contributed to these observations as confounding factors.^18^ Therefore, administering the full vaccination schedule to a single portion of the population would have a positive effect compared to administering only one dose to a more significant portion of the population in the context of the CoronaVac immunizer.^15^

The effectiveness of CoronaVac among healthcare professionals showed 50.7 %, 83.7 %, and 100 % efficacy in symptomatic infections, hospitalizations, and severe cases, respectively.^16^ Other studies showed 47 % effectiveness in preventing symptomatic infections and severity in the population ≥ 70-years-old (61 % effectiveness in preventing cases of death and 55 % for hospitalizations).^13^ A study examining the Latin American population showed 33 % effectiveness among partially vaccinated individuals, while among those fully vaccinated, a 53 % effectiveness was found.^9^

This study had some limitations due to its retrospective nature and the critical period of the pandemic, which may have resulted in the potential loss of clinical data. However, it contributes by providing clinical, epidemiological, and even phylogenetic information from COVID-19 patients hospitalized during the pandemic. Furthermore, this analysis highlights the critical importance of ongoing assessments regarding the impact of decisions on implementing preventive measures on public health, which can help with future vaccination strategies.

Since the new coronavirus was described in early 2020, there has been a major effort to develop vaccines that enhance immunity against the new agent. However, after the COVID-19 vaccines were developed, the circulating lineage corresponded to the ancestral variant, and only approximately a year later, the vaccines started to be administered to the population. Additional variants were described, which had a crucial role in sustaining the pandemic through point mutations that conferred immune response escape and high rates of infection and transmissibility.^16^^,^^19^

In conclusion, this study found no evidence of vaccination affecting the survival of hospitalized patients in this cohort. However, the results are specific to a tertiary care hospital, where patients generally present with more severe cases, multiple comorbidities, and higher intensive care requirements, limiting the generalizability to less severe cases. Therefore, it is crucial to highlight that these findings should not be extrapolated, especially given the clear and undeniable benefits vaccines have shown in controlling the pandemic. Continuous surveillance of circulating strains, such as through the sentinel surveillance network for influenza and other respiratory virus infections, and ongoing research on vaccinated individuals remain crucial for generating data to inform public health policies.

## CRediT authorship contribution statement

**Laura Holtman-Ferreira:** Formal analysis, Investigation, Writing – original draft. **Elessandra de Souza Bitencourt:** Funding acquisition, Project administration. **Betina Mendez de Alcantara Gabardo:** Data curation, Formal analysis, Investigation, Methodology. **Susanne Edinger Pereira:** Data curation, Formal analysis, Investigation, Methodology. **Francine Teixeira:** Data curation, Formal analysis, Investigation, Methodology. **Diego da Silva Magatão:** Data curation, Formal analysis, Investigation, Methodology. **Vitor Loureiro Dias:** Data curation, Formal analysis, Investigation, Methodology. **Ricardo Petterle:** Data curation. **Meri Bordignon Nogueira:** Funding acquisition, Investigation. **Sonia Mara Raboni:** Conceptualization, Data curation, Formal analysis, Funding acquisition, Investigation, Methodology, Project administration, Resources, Supervision, Validation, Writing – review & editing.

## Conflicts of interest

The authors declare no conflicts of interests.

## Appendix Supplementary materials

Image, application 1
