# Supplementary material for: COVID-19 hospitalization in vaccinated and non-vaccinated patients: Clinical profile and outcomes
Source: Braz J Infect Dis. 2025 May 9;29(3):104537. doi: 10.1016/j.bjid.2025.104537 (PMC12146633; doi:10.1016/j.bjid.2025.104537)
Supplement: Supplementary file 1 [file mmc1.docx]

BJID-D-24-00292_ **Supplementary Material**

**Supplementary Material** GISAID ID of sequenced SARS-CoV-2 viruses.

| **Number** | **GISAID ID** |
| --- | --- |
| 1 | hCoV-19/Brazil/PR-FIOCRUZ-13474/2022 |
| 2 | hCoV-19/Brazil/PR-FIOCRUZ-13475/2022 |
| 3 | hCoV-19/Brazil/PR-FIOCRUZ-13476/2022 |
| 4 | hCoV-19/Brazil/PR-FIOCRUZ-13477/2022 |
| 5 | hCoV-19/Brazil/PR-FIOCRUZ-13478/2022 |
| 6 | hCoV-19/Brazil/PR-FIOCRUZ-13479/2022 |
| 7 | hCoV-19/Brazil/PR-FIOCRUZ-13480/2022 |
| 8 | hCoV-19/Brazil/PR-FIOCRUZ-13481/2022 |
| 9 | hCoV-19/Brazil/PR-FIOCRUZ-13482/2022 |
| 10 | hCoV-19/Brazil/PR-FIOCRUZ-13483/2022 |
| 11 | hCoV-19/Brazil/PR-FIOCRUZ-13484/2022 |
| 12 | hCoV-19/Brazil/PR-FIOCRUZ-13485/2022 |
| 13 | hCoV-19/Brazil/PR-FIOCRUZ-13486/2022 |
| 14 | hCoV-19/Brazil/PR-FIOCRUZ-13487/2022 |
| 15 | hCoV-19/Brazil/PR-FIOCRUZ-13488/2022 |
| 16 | hCoV-19/Brazil/PR-FIOCRUZ-13489/2022 |
| 17 | hCoV-19/Brazil/PR-FIOCRUZ-13490/2022 |
| 18 | hCoV-19/Brazil/PR-FIOCRUZ-13492/2022 |
| 19 | hCoV-19/Brazil/PR-FIOCRUZ-13493/2022 |
| 20 | hCoV-19/Brazil/PR-FIOCRUZ-13494/2022 |
| 21 | hCoV-19/Brazil/PR-FIOCRUZ-13495/2022 |
| 22 | hCoV-19/Brazil/PR-FIOCRUZ-13499/2022 |
| 23 | hCoV-19/Brazil/PR-FIOCRUZ-13501/2022 |
| 24 | hCoV-19/Brazil/PR-FIOCRUZ-13502/2022 |
| 25 | hCoV-19/Brazil/PR-FIOCRUZ-13503/2022 |
| 26 | hCoV-19/Brazil/PR-FIOCRUZ-34554/2020 |
| 27 | hCoV-19/Brazil/PR-FIOCRUZ-34555/2020 |
| 28 | hCoV-19/Brazil/PR-FIOCRUZ-34556/2020 |
| 29 | hCoV-19/Brazil/PR-FIOCRUZ-34557/2020 |
| 30 | hCoV-19/Brazil/PR-FIOCRUZ-34558/2020 |
| 31 | hCoV-19/Brazil/PR-FIOCRUZ-34559/2020 |
| 32 | hCoV-19/Brazil/PR-FIOCRUZ-34560/2020 |
| 33 | hCoV-19/Brazil/PR-FIOCRUZ-34562/2020 |
| 34 | hCoV-19/Brazil/PR-FIOCRUZ-34565/2020 |
| 35 | hCoV-19/Brazil/PR-FIOCRUZ-34566/2020 |
| 36 | hCoV-19/Brazil/PR-FIOCRUZ-68433/2021 |
| 37 | hCoV-19/Brazil/PR-FIOCRUZ-68434/2021 |
| 38 | hCoV-19/Brazil/PR-FIOCRUZ-68435/2021 |
| 39 | hCoV-19/Brazil/PR-FIOCRUZ-68437/2021 |
| 40 | hCoV-19/Brazil/PR-FIOCRUZ-68438/2021 |
| 41 | hCoV-19/Brazil/PR-FIOCRUZ-68439/2021 |
| 42 | hCoV-19/Brazil/PR-FIOCRUZ-68440/2021 |
| 43 | hCoV-19/Brazil/PR-FIOCRUZ-68441/2021 |
| 44 | hCoV-19/Brazil/PR-FIOCRUZ-68442/2021 |
| 45 | hCoV-19/Brazil/PR-FIOCRUZ-68445/2021 |
| 46 | hCoV-19/Brazil/PR-FIOCRUZ-68289/2021 |
| 47 | hCoV-19/Brazil/PR-FIOCRUZ-68290/2021 |
| 48 | hCoV-19/Brazil/PR-FIOCRUZ-68291/2021 |
| 49 | hCoV-19/Brazil/PR-FIOCRUZ-68292/2021 |
| 50 | hCoV-19/Brazil/PR-FIOCRUZ-68293/2021 |
| 51 | hCoV-19/Brazil/PR-FIOCRUZ-68294/2021 |
| 52 | hCoV-19/Brazil/PR-FIOCRUZ-68295/2021 |
| 53 | hCoV-19/Brazil/PR-FIOCRUZ-68296/2021 |
| 54 | hCoV-19/Brazil/PR-FIOCRUZ-68298/2021 |
| 55 | hCoV-19/Brazil/PR-FIOCRUZ-68299/2021 |
| 56 | hCoV-19/Brazil/PR-FIOCRUZ-68303/2021 |
| 57 | hCoV-19/Brazil/PR-FIOCRUZ-68305/2021 |
| 58 | hCoV-19/Brazil/PR-FIOCRUZ-68306/2021 |
| 59 | hCoV-19/Brazil/PR-FIOCRUZ-68308/2021 |
| 60 | hCoV-19/Brazil/PR-FIOCRUZ-68310/2021 |
| 61 | hCoV-19/Brazil/PR-FIOCRUZ-68311/2021 |
| 62 | hCoV-19/Brazil/PR-FIOCRUZ-68313/2021 |
| 63 | hCoV-19/Brazil/PR-FIOCRUZ-68315/2021 |
| 64 | hCoV-19/Brazil/PR-FIOCRUZ-68316/2021 |
| 65 | hCoV-19/Brazil/PR-FIOCRUZ-68318/2021 |
| 66 | hCoV-19/Brazil/PR-FIOCRUZ-68322/2021 |
| 67 | hCoV-19/Brazil/PR-FIOCRUZ-68323/2021 |
| 68 | hCoV-19/Brazil/PR-FIOCRUZ-68327/2021 |
| 69 | hCoV-19/Brazil/PR-FIOCRUZ-68328/2021 |
| 70 | hCoV-19/Brazil/PR-FIOCRUZ-68329/2021 |
| 71 | hCoV-19/Brazil/PR-FIOCRUZ-68330/2021 |
| 72 | hCoV-19/Brazil/PR-FIOCRUZ-68331/2021 |
| 73 | hCoV-19/Brazil/PR-FIOCRUZ-68333/2021 |
| 74 | hCoV-19/Brazil/PR-FIOCRUZ-68335/2021 |
| 75 | hCoV-19/Brazil/PR-FIOCRUZ-68336/2021 |
| 76 | hCoV-19/Brazil/PR-FIOCRUZ-68337/2021 |
| 77 | hCoV-19/Brazil/PR-FIOCRUZ-68338/2021 |
| 78 | hCoV-19/Brazil/PR-FIOCRUZ-68339/2021 |
| 79 | hCoV-19/Brazil/PR-FIOCRUZ-68341/2021 |
| 80 | hCoV-19/Brazil/PR-FIOCRUZ-68342/2021 |
| 81 | hCoV-19/Brazil/PR-FIOCRUZ-68343/2021 |
| 82 | hCoV-19/Brazil/PR-FIOCRUZ-68344/2021 |
| 83 | hCoV-19/Brazil/PR-FIOCRUZ-68348/2021 |
| 84 | hCoV-19/Brazil/PR-FIOCRUZ-68351/2021 |
| 85 | hCoV-19/Brazil/PR-FIOCRUZ-68352/2021 |
| 86 | hCoV-19/Brazil/PR-FIOCRUZ-68353/2021 |
| 87 | hCoV-19/Brazil/PR-FIOCRUZ-68354/2021 |
| 88 | hCoV-19/Brazil/PR-FIOCRUZ-68355/2021 |
| 89 | hCoV-19/Brazil/PR-FIOCRUZ-68357/2021 |
| 90 | hCoV-19/Brazil/PR-FIOCRUZ-68359/2021 |
| 91 | hCoV-19/Brazil/PR-FIOCRUZ-68360/2021 |
| 92 | hCoV-19/Brazil/PR-FIOCRUZ-68361/2021 |
| 93 | hCoV-19/Brazil/PR-FIOCRUZ-68362/2021 |
| 94 | hCoV-19/Brazil/PR-FIOCRUZ-68367/2021 |
| 95 | hCoV-19/Brazil/PR-FIOCRUZ-68368/2021 |
| 96 | hCoV-19/Brazil/PR-FIOCRUZ-68369/2021 |
| 97 | hCoV-19/Brazil/PR-FIOCRUZ-68370/2021 |
| 98 | hCoV-19/Brazil/PR-FIOCRUZ-68371/2021 |
| 99 | hCoV-19/Brazil/PR-FIOCRUZ-68373/2021 |
| 100 | hCoV-19/Brazil/PR-FIOCRUZ-68374/2021 |
| 101 | hCoV-19/Brazil/PR-FIOCRUZ-68375/2021 |
| 102 | hCoV-19/Brazil/PR-FIOCRUZ-68376/2021 |
| 103 | hCoV-19/Brazil/PR-FIOCRUZ-68377/2021 |
| 104 | hCoV-19/Brazil/PR-FIOCRUZ-68378/2021 |
| 105 | hCoV-19/Brazil/PR-FIOCRUZ-68379/2021 |
| 106 | hCoV-19/Brazil/PR-FIOCRUZ-68380/2021 |
| 107 | hCoV-19/Brazil/PR-FIOCRUZ-68381/2021 |
| 108 | hCoV-19/Brazil/PR-FIOCRUZ-68382/2021 |
| 109 | hCoV-19/Brazil/PR-FIOCRUZ-68384/2021 |
| 110 | hCoV-19/Brazil/PR-FIOCRUZ-68385/2021 |
| 111 | hCoV-19/Brazil/PR-FIOCRUZ-68386/2021 |
| 112 | hCoV-19/Brazil/PR-FIOCRUZ-68387/2021 |
| 113 | hCoV-19/Brazil/PR-FIOCRUZ-68389/2021 |
| 114 | hCoV-19/Brazil/PR-FIOCRUZ-68390/2021 |
| 115 | hCoV-19/Brazil/PR-FIOCRUZ-68391/2021 |
| 116 | hCoV-19/Brazil/PR-FIOCRUZ-68392/2021 |
| 117 | hCoV-19/Brazil/PR-FIOCRUZ-68393/2021 |
| 118 | hCoV-19/Brazil/PR-FIOCRUZ-68394/2021 |
| 119 | hCoV-19/Brazil/PR-FIOCRUZ-68395/2021 |
| 120 | hCoV-19/Brazil/PR-FIOCRUZ-68396/2021 |
| 121 | hCoV-19/Brazil/PR-FIOCRUZ-68398/2021 |
| 122 | hCoV-19/Brazil/PR-FIOCRUZ-68399/2021 |
| 123 | hCoV-19/Brazil/PR-FIOCRUZ-68400/2021 |
| 124 | hCoV-19/Brazil/PR-FIOCRUZ-68401/2021 |
| 125 | hCoV-19/Brazil/PR-FIOCRUZ-68402/2021 |
| 126 | hCoV-19/Brazil/PR-FIOCRUZ-68403/2021 |
| 127 | hCoV-19/Brazil/PR-FIOCRUZ-68404/2021 |
| 128 | hCoV-19/Brazil/PR-FIOCRUZ-68405/2021 |
| 129 | hCoV-19/Brazil/PR-FIOCRUZ-68408/2021 |
| 130 | hCoV-19/Brazil/PR-FIOCRUZ-68409/2021 |
| 131 | hCoV-19/Brazil/PR-FIOCRUZ-68412/2021 |
| 132 | hCoV-19/Brazil/PR-FIOCRUZ-68413/2021 |
| 133 | hCoV-19/Brazil/PR-FIOCRUZ-68414/2021 |
| 134 | hCoV-19/Brazil/PR-FIOCRUZ-68416/2021 |
| 135 | hCoV-19/Brazil/PR-FIOCRUZ-68417/2021 |
| 136 | hCoV-19/Brazil/PR-FIOCRUZ-68420/2021 |
| 137 | hCoV-19/Brazil/PR-FIOCRUZ-68422/2021 |
| 138 | hCoV-19/Brazil/PR-FIOCRUZ-68426/2021 |
| 139 | hCoV-19/Brazil/PR-FIOCRUZ-68428/2021 |
| 140 | hCoV-19/Brazil/PR-FIOCRUZ-68429/2021 |
| 141 | hCoV-19/Brazil/PR-FIOCRUZ-59669/2021 |
| 142 | hCoV-19/Brazil/PR-FIOCRUZ-59616/2021 |
| 143 | hCoV-19/Brazil/PR-FIOCRUZ-59648/2021 |
| 144 | hCoV-19/Brazil/PR-FIOCRUZ-59629/2021 |
| 145 | hCoV-19/Brazil/PR-FIOCRUZ-59630/2021 |
| 146 | hCoV-19/Brazil/PR-FIOCRUZ-59673/2021 |
| 147 | hCoV-19/Brazil/PR-FIOCRUZ-59631/2021 |
| 148 | hCoV-19/Brazil/PR-FIOCRUZ-59667/2021 |
| 149 | hCoV-19/Brazil/PR-FIOCRUZ-59657/2021 |
| 150 | hCoV-19/Brazil/PR-FIOCRUZ-59668/2021 |
| 151 | hCoV-19/Brazil/PR-FIOCRUZ-59617/2021 |
| 152 | hCoV-19/Brazil/PR-FIOCRUZ-59632/2021 |
| 153 | hCoV-19/Brazil/PR-FIOCRUZ-59624/2021 |
| 154 | hCoV-19/Brazil/PR-FIOCRUZ-59644/2021 |
| 155 | hCoV-19/Brazil/PR-FIOCRUZ-59627/2021 |
| 156 | hCoV-19/Brazil/PR-FIOCRUZ-59651/2021 |
| 157 | hCoV-19/Brazil/PR-FIOCRUZ-59661/2021 |
| 158 | hCoV-19/Brazil/PR-FIOCRUZ-59638/2021 |
| 159 | hCoV-19/Brazil/PR-FIOCRUZ-59647/2021 |
| 160 | hCoV-19/Brazil/PR-FIOCRUZ-59589/2021 |
| 161 | hCoV-19/Brazil/PR-FIOCRUZ-59602/2021 |
| 162 | hCoV-19/Brazil/PR-FIOCRUZ-59609/2021 |
| 163 | hCoV-19/Brazil/PR-FIOCRUZ-59637/2021 |
| 164 | hCoV-19/Brazil/PR-FIOCRUZ-59670/2021 |
| 165 | hCoV-19/Brazil/PR-FIOCRUZ-59598/2021 |
| 166 | hCoV-19/Brazil/PR-FIOCRUZ-59640/2021 |
| 167 | hCoV-19/Brazil/PR-FIOCRUZ-59618/2021 |
| 168 | hCoV-19/Brazil/PR-FIOCRUZ-59650/2021 |
| 169 | hCoV-19/Brazil/PR-FIOCRUZ-59639/2021 |
| 170 | hCoV-19/Brazil/PR-FIOCRUZ-59655/2021 |
| 171 | hCoV-19/Brazil/PR-FIOCRUZ-59587/2021 |
| 172 | hCoV-19/Brazil/PR-FIOCRUZ-59603/2021 |
| 173 | hCoV-19/Brazil/PR-FIOCRUZ-59591/2021 |
| 174 | hCoV-19/Brazil/PR-FIOCRUZ-59621/2021 |
| 175 | hCoV-19/Brazil/PR-FIOCRUZ-59663/2021 |
| 176 | hCoV-19/Brazil/PR-FIOCRUZ-59604/2021 |
| 177 | hCoV-19/Brazil/PR-FIOCRUZ-59659/2021 |
| 178 | hCoV-19/Brazil/PR-FIOCRUZ-59606/2021 |
